# Rufomycin Exhibits Dual Effects Against *Mycobacterium abscessus* Infection by Inducing Host Defense and Antimicrobial Activities

**DOI:** 10.3389/fmicb.2021.695024

**Published:** 2021-08-10

**Authors:** Cho Rong Park, Seungwha Paik, Young Jae Kim, Jin Kyung Kim, Sang Min Jeon, Sang-Hee Lee, Jake Whang, Jinhua Cheng, Joo-Won Suh, Jin Cao, Gauri Shetye, Shao-Nong Chen, James McAlpine, Guido F. Pauli, Scott Franzblau, Sanghyun Cho, Eun-Kyeong Jo

**Affiliations:** ^1^Department of Microbiology, Chungnam National University School of Medicine, Daejeon, South Korea; ^2^Department of Medical Science, Chungnam National University School of Medicine, Daejeon, South Korea; ^3^Infection Control Convergence Research Center, Chungnam National University School of Medicine, Daejeon, South Korea; ^4^Center for Research Equipment, Korea Basic Science Institute, Cheongju, South Korea; ^5^Korea Mycobacterium Resource Center & Basic Research Section, The Korean Institute of Tuberculosis, Cheongju, South Korea; ^6^Myongji Bioefficacy Research Center, Myongji University, Yongin, South Korea; ^7^Institute for Tuberculosis Research, College of Pharmacy, University of Illinois at Chicago, Chicago, IL, United States; ^8^Department of Pharmaceutical Sciences, College of Pharmacy, University of Illinois at Chicago, Chicago, IL, United States

**Keywords:** *Mycobacterium abscessus*, rufomycin, inflammation, host immune defense, antimicrobial activity

## Abstract

Nontuberculous mycobacterial pulmonary infection is often aggravated due to antibiotic resistance issues. There is a need for development of new drugs inducing both host immune responses and antimicrobial activities. This study shows that the rufomycins 4/5/6/7 (Rufomycin 4–7), which targets ClpC1 as a subunit of caseinolytic protein complex ClpC1/ClpP1/ClpP2 of mycobacteria, exhibits a dual effect in host innate defense and *in vivo* antimicrobial activities against a rough morphotype of *Mycobacterium abscessus* (Mabs-R), a clinically severe morphotype that causes hyperinflammation. Rufomycin 4–7 treatment showed antimicrobial effects against Mabs pulmonary infection *in vivo* and in macrophages. In addition, Rufomycin 4–7 significantly decreased inflammation, but enhanced the autophagy/lysosomal genes through upregulation of the nuclear translocation of transcription factor EB (TFEB). Furthermore, Rufomycin 4–7 treatment effectively inhibited mitochondrial damage and oxidative stresses in macrophages during Mabs-R infection. Collectively, Rufomycin 4–7-mediated dual effects inducing both antimicrobial activities and host immune defense might confer an advantage to treatment against Mabs-R infection.

## Introduction

Nontuberculous mycobacteria (NTM) are a diverse group of more than 190 bacilli, other than *Mycobacterium tuberculosis* complex (Porvaznik et al., 2017). Although NTM are ubiquitously found in the environments, some of them are able to cause disease in immunocompetent and immunocompromised persons (Simons et al., 2011; Wassilew et al., 2016; Porvaznik et al., 2017; Maiz Carro et al., 2018; Sethiya et al., 2020). The prevalence and incidence of NTM diseases are increasing, thus having emerged as important pathogens worldwide (Wassilew et al., 2016; To et al., 2020). NTM can cause a wide range of human infections, and the most common clinical feature is a chronic pulmonary infection (Simons et al., 2011; Wassilew et al., 2016; Maiz Carro et al., 2018; To et al., 2020). Among rapidly growing NTM, *Mycobacterium abscessus* subsp. *abscessus* (Mabs) is the most worrisome pathogen and difficult to treat due to high resistance to many antibiotics (Luthra et al., 2018; Strnad and Winthrop, 2018; Degiacomi et al., 2019). There is an urgent need for novel host-directed therapeutic approaches that boost host defense pathways as well as target Mabs pathogens, thereby decreasing the prospect of pathogens developing resistance during treatment.

Mabs are divided into two colony morphology variants, such as a smooth-colony variant (Mabs-S) that possesses glycopeptidolipid (GPL) which is crucial for environmental colonization and associated with biofilm formation, and a rough-colony variant (Mabs-R), which lacks GPL (Howard et al., 2006; Pawlik et al., 2013). Mabs-R morphotypes are often associated with more severe clinical manifestations with persistent infections (Howard et al., 2006; Pawlik et al., 2013). Mabs-R exhibits an invasive phenotype and efficiently activates the innate and inflammatory immune responses in human and mouse macrophages, whereas Mabs-S does little (Rhoades et al., 2009). Earlier studies showed that Mabs-R resulted in more neutrophil infiltration into bronchoalveolar lavage fluids than Mabs-S did in mice (Caverly et al., 2015). In human peripheral blood mononuclear cells, Mabs-R induced more IL-1β, but less IL-10, than Mabs-S did (Jonsson et al., 2013). In addition, Mabs-R strains robustly activated inflammatory responses and NLRP3 inflammasomes through induction of mitochondrial reactive oxygen species (mtROS) in macrophages (Kim B. R. et al., 2020). Mabs-R also accesses cytosols through phagosomal rupture, thereby promoting type I interferon (IFN) release, cell death, and cell-to-cell spreading (Kim B. R. et al., 2019). More recently, Mabs-R-susceptible sirtuin 3 mice induced mitochondrial damage and pathological inflammation during infection (Kim Y. J. et al., 2020). These studies enabled the investigation of a new strategy for anti-Mabs drugs that employ a bidirectional mode, not only through antimicrobial activity but also *via* host immune modulation.

Rufomycins 4/5/6/7, the complex formed by Rufomycins 4–7 (called Rufomycin 4–7 in the following), is the currently known most potent rufomycin produced by *Streptomyces atratus* and represents a difficult-to-purify complex of four partially interconvertible diastereomers. Rufomycin 4–7 is known to target the ClpC1, which is a subunit of caseinolytic protein complex ClpC1/ClpP1/ClpP2, thereby inhibiting intracellular growth of mycobacteria including Mabs-S (Choules et al., 2019). However, its effects upon Mabs-R infection have not been studied. This study investigated how Rufomycin 4–7 induces antimicrobial effects against Mabs-R pulmonary infection *in vivo* and in macrophages. Moreover, Rufomycin 4–7 had an effect of controlling pathological inflammation, increasing autophagy and lysosomal gene expression, and thereby downregulating mitochondrial damage during Mabs-R infection. These results support that Rufomycin 4–7 is a potential hit lead compound against Mabs through a dual mode of action inducing antimicrobial responses and host immune defense.

## Results

### Antimicrobial Effects of Rufomycin 4–7 Against Mabs-R Infection

**FIGURE 1:**
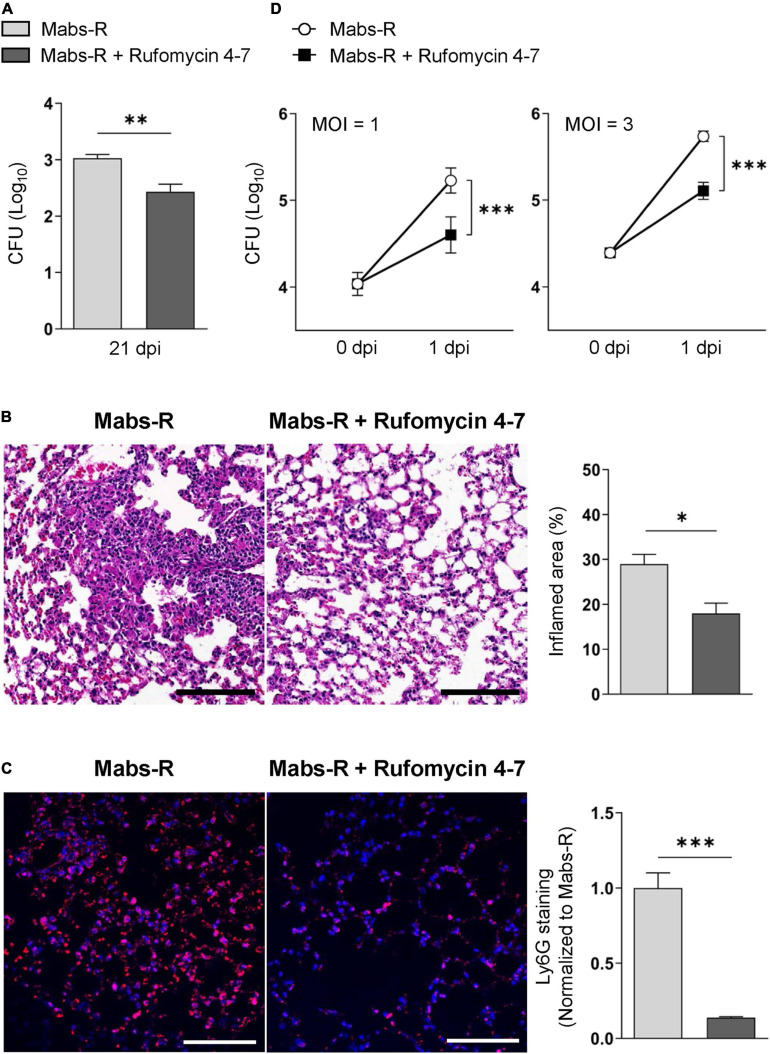
Rufomycin 4–7 treatment increases antimicrobial effects against mycobacterial infection *in vivo* and *in vitro*. **(A)** Mice were infected with Mabs-R (1 × 10^6^ CFU) intranasally, followed by administration of Rufomycin 4–7 (200 mg/kg, three times per week; by gavage) and sacrificed at 21 dpi. Bacterial loads in lung tissues for infected mice (*n* = 5; each group) were determined by CFU assay. **(B)** The sectioned lung tissues of infected mice (*n* = 3; each group) from panel **(A)** were H&E stained (scale bar: 300 μm), and the inflamed area of each group was quantified. **(C)** The sectioned lung tissues from panel **(A)** were stained with anti-Ly6G (red) and DAPI (blue) (scale bar: 25 μm), and the relative fluorescence intensity of Ly6G-stained neutrophils were quantified (*n* = 9 for each group; at least 200 cells per image). **(D)** BMDMs were infected with Mabs-R (MOI = 1 or 3) for 2 h and then incubated with solvent control (sc) or Rufomycin 4–7 (10 μM) in the freshly changed media. Intracellular survival of Mabs-R was determined by CFU assay at 0 and 1 dpi. ^∗^*p* < 0.05, ^∗∗^*p* < 0.01, and ^∗∗∗^*p* < 0.001. Statistical analysis was determined with unpaired *t*-test **(A–C)** or two-way ANOVA **(D)**. Data are presented as means ± SEM **(A–C)**, or ± SD from at least three independent experiments performed in triplicate **(D)**. Images are representative of three independent experiments **(B,C)**. MOI, multiplicity of infection; dpi, days post-infection.

In the previous study, Rufomycin 4–7 showed potent inhibitory effects against both *M. tuberculosis* (Mtb) [minimal inhibitory concentration (MIC), 0.02 μM] and Mabs-S (MIC, 0.4 μM) (Choules et al., 2019). On the other hand, the MIC of Rufomycin 4–7 against several Mabs-R strains was assessed as 0.5–4 μg/ml (Supplementary Table 1), which was less effective than in inhibiting Mabs-S. However, to evaluate its antimicrobial effects *in vivo*, Rufomycin 4–7 was administered to the Mabs-R-infected mice. By using the Mabs-R pulmonary infection model established in the previous work (Kim Y. J. et al., 2020), it was confirmed that Rufomycin 4–7 treatment significantly decreased Mabs-R survival in the mouse lungs at 21 days post-infection (dpi) (Figure 1A). In addition, Rufomycin 4–7 administration significantly inhibited Mabs-R-mediated inflammatory reactions in the mouse lungs. The damaged histologic structure of lung tissues from the infected mice showed that the total inflamed area of the lungs was significantly decreased upon Rufomycin 4–7 administration (Figure 1B). In addition, Ly6G-stained neutrophils were also significantly diminished in the lungs of the Mabs-R-infected mice (Figure 1C) with Rufomycin 4-7 treatment.

To further investigate the effects of Rufomycin 4–7 against Mabs-R *in vitro*, intracellular survival of Mabs-R was assessed in bone marrow-derived macrophages (BMDMs). As shown in Figure 1D, intracellular growth rates of Mabs-R were significantly decreased in BMDMs by Rufomycin 4–7 treatment. Similar to these findings, intracellular survival of the rough strain of *M. massiliense* (Mmass-R) was also reduced by treatment of Rufomycin 4–7 (Supplementary Figure 1). Moreover, intracellular survival of clinical isolate KMRC-00800-00018 (#18) and KMRC-00800-00019 (#19) of Mabs-R was also significantly reduced with Rufomycin 4–7 treatment in BMDMs (Supplementary Figure 2). Together, these data suggest that Rufomycin 4–7 has an antimicrobial activity against Mabs-R infection *in vitro* and *in vivo*.

### Rufomycin 4–7 Ameliorates Mabs-R-Induced Exaggerated Inflammatory Cytokine Production in Macrophages

**FIGURE 2:**
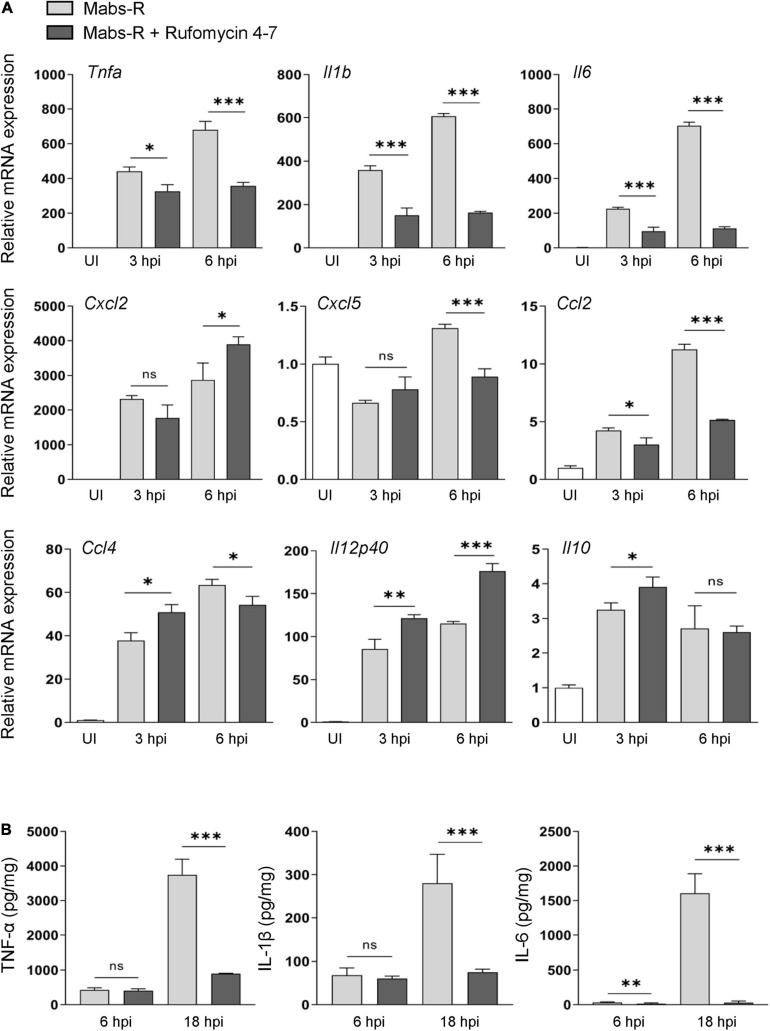
Rufomycin 4–7 regulates the expression of inflammatory cytokines during Mabs-R infection. **(A)** BMDMs were infected with Mabs-R (MOI = 3) for 2 h and then incubated with sc or Rufomycin 4–7 (10 μM) in the freshly changed media. The cells were harvested at the indicated times and subjected to qRT-PCR analysis to measure the expression of inflammatory cytokine/chemokine genes. **(B)** The supernatants from the BMDMs prepared as in **(A)** were collected at 6 and 18 hpi and subjected to ELISA to measure the cytokine level of TNF-α, IL-1β, and IL-6. ^∗^*p* < 0.05, ^∗∗^*p* < 0.01, and ^∗∗∗^*p* < 0.001. Statistical analysis was determined with unpaired *t*-test and presented as means ± SD from at least three independent experiments performed in triplicate. ns, not significant; UI, uninfected; hpi, hours post-infection.

A previous study showed that exaggerated pathologic inflammation played a detrimental role in antimicrobial host defense against Mabs-R infection (Kim Y. J. et al., 2020). To explore whether Rufomycin 4–7 ameliorated inflammatory responses and promoted protective immune functions, the relative expressions of cytokines and chemokines were examined in the Mabs-infected BMDMs. It was found that Rufomycin 4–7 treatment significantly decreased the Mabs-R-induced gene expression of various proinflammatory cytokines and chemokines (*Tnfa*, *Il1b*, *Il6*, *Cxcl5*, *Ccl2*, and *Ccl4*) in BMDMs at 6 h post-infection (hpi) (Figure 2A). However, the mRNA expression levels of Mabs-R-induced *Cxcl2* and *Il12p40*, which were involved in the induction of Th1 and Th17 lymphocytes (Kuwabara et al., 2017), respectively, were substantially increased in BMDMs by treatment with Rufomycin 4–7 at 6 hpi (Figure 2A). In addition, there was no significant difference in Mabs-induced *Il10* mRNA expression in BMDMs between the presence and absence of Rufomycin 4–7 at 6 hpi (Figure 2A). It was also shown that Rufomycin 4–7 treatment significantly reduced the *Il1b* and *Il6* expression induced by the clinical isolates #18 and #19 in BMDMs at both 3 and 6 hpi (Supplementary Figure 3).

Next, enzyme-linked immunosorbent assay (ELISA) was performed to verify whether Rufomycin 4–7 regulated the protein levels of cytokines in Mabs-infected BMDMs. Mabs infection robustly increased the production of proinflammatory cytokines including TNF-α, IL-6, and IL-1β in BMDMs at 18 hpi (Figure 2B). In accordance with the data of gene expression, Rufomycin 4–7 treatment dramatically decreased the Mabs-R-induced production of TNF-α, IL-6, and IL-1β, in BMDMs (Figure 2B). These results suggest that Rufomycin 4–7 reduces a number of damaging proinflammatory cytokines/chemokines in macrophages during Mabs-R infection.

### Rufomycin 4–7 Suppresses Mabs-R-Triggered p38-MAPK Inflammatory Signaling in Macrophages

**FIGURE 3:**
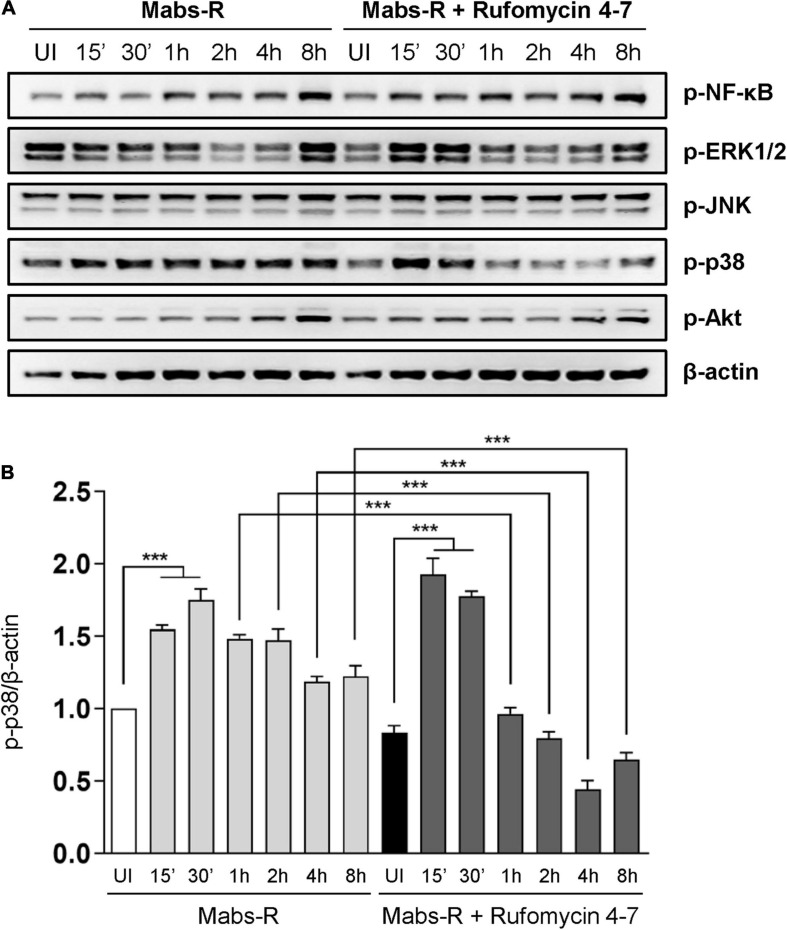
Rufomycin 4–7 decreases Mabs-R-induced p38-MAPK inflammatory signaling in macrophages. **(A)** BMDMs were pretreated with Rufomycin 4–7 (10 μM) or vehicle and infected with Mabs-R (MOI = 3) for the indicated times. Cells were lysed and subjected to immunoblot analysis with antibodies of p-NF-κB, p-ERK, p-JNK, p-p38, p-Akt, and β-actin. **(B)** Densitometric analysis was performed and normalized to β-actin with the p-p38 band. ***p < 0.001. Statistical analysis was determined with one-way ANOVA and presented as means ± SD **(B)**. Western blot images are representative of three independent experiments **(A)**. UI, uninfected.

To study the mechanisms by which Rufomycin 4–7 regulates inflammatory signaling activation, the phosphorylation state of nuclear factor (NF)-κB or mitogen-activated protein kinases (MAPKs) required for innate immune signaling during mycobacterial and NTM infection (Schorey and Cooper, 2003; Sampaio et al., 2008; Kim et al., 2014) was assessed (Figure 3A). Among the MAPKs, phospho-p38-MAPK (p-p38) was significantly downregulated with Rufomycin 4–7 treatment. Specifically, Mabs-R significantly triggered the p-p38 in BMDMs 15–30 min after infection, but Rufomycin 4–7 significantly decreased the p-p38 level at 1–8 hpi (Figure 3B). However, Rufomycin 4–7 did not modulate the activation of other signaling pathways such as phospho-NF-κB (p-NF-κB), phospho-extracellular signal-regulated kinase 1/2 (p-ERK1/2), phospho-c-Jun N-terminal kinase (p-JNK), or phospho-Akt/protein kinase B (p-Akt) in BMDMs during Mabs-R infection (Figure 3B). These results suggest that Rufomycin 4–7 selectively inhibits the Mabs-R-induced p-p38-MAPK, which may principally contribute to inflammatory responses, in BMDMs.

### Rufomycin 4–7 Upregulates TFEB-Lysosomal Gene Expression and Colocalization of Phagosomes and Lysosomes During Mabs Infection

**FIGURE 4:**
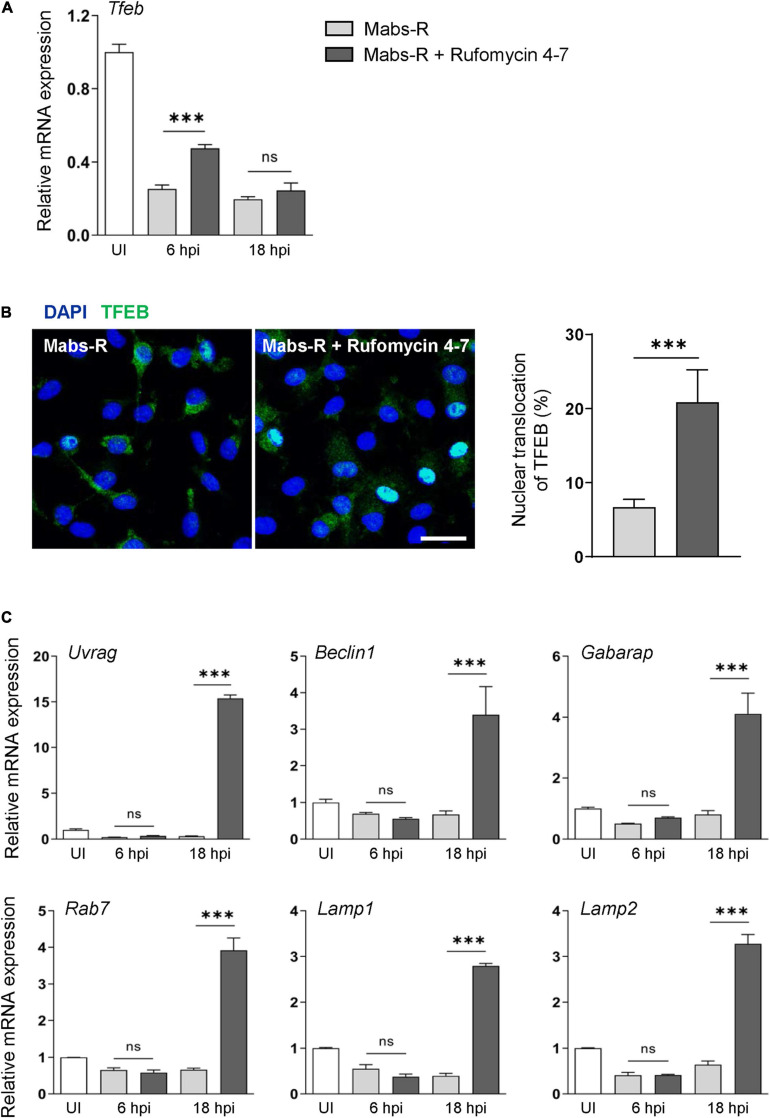
Rufomycin 4–7 enhances TFEB-lysosomal gene expression during Mabs-R infection. BMDMs were infected with Mabs-R (MOI = 3) for 2 h and then incubated with sc or Rufomycin 4–7 (10 μM) in the freshly changed media for the designated time. **(A)** The mRNA expression level of TFEB was determined by qRT-PCR. **(B)** At 12 hpi, the cells were fixed, permed, and stained with anti-TFEB antibodies (green) and DAPI (blue) to visualize fluorescent images using confocal microscopy (scale bar: 12.5 μm). The cells with TFEB translocated into the nucleus was manually calculated from the confocal images (*n* = 8 for each group; at least 80 cells per image). **(C)** Cells were lysed and total RNAs were extracted, followed by qRT-PCR analysis with primers of TFEB-downstream autophagic/lysosomal genes including *Uvrag*, *Beclin1*, *Gabarap*, *Rab7*, *Lamp1*, and *Lamp2.* ^∗∗∗^*p* < 0.001. Statistical analysis was determined with one-way ANOVA **(A,C)** or unpaired *t*-test **(B)**. Data are presented as means ± SD from at least three independent experiments performed in triplicate. ns, not significant; UI, uninfected; hpi, hours post-infection.

It was reported that transcription factor EB (TFEB) expression is critically required for host innate defense against Mabs infection (Kim Y. S. et al., 2020). Thus, it was questioned how *Tfeb* expression is modulated in BMDMs by either Mabs-R infection or Rufomycin 4–7 treatment. As shown in Figure 4A, Mabs-R infection led to a marked inhibition of *Tfeb* gene expression in BMDMs, and Rufomycin 4–7 partly recovered the *Tfeb* expression after 6 h of treatment. Then, further examination was performed to find whether Rufomycin 4–7 led to an increase of nuclear translocation of TFEB in Mabs-R-infected BMDMs. When the kinetics of TFEB nuclear translocation was assessed in a time-dependent manner, Rufomycin 4–7 treatment significantly upregulated the nuclear translocation of TFEB in Mabs-R-infected BMDMs at 12 hpi (Figure 4B). It was then analyzed whether Rufomycin 4–7 activated the TFEB-downstream autophagic/lysosomal genes in Mabs-R-infected BMDMs. The mRNA expression levels of TFEB downstream genes including *Uvrag*, *Beclin1*, *Gabarap*, and *Rab7* were markedly upregulated by Rufomycin 4–7 in Mabs-R-infected BMDMs (Figure 4C). In addition, Rufomycin 4–7 treatment led to a significant increase of Mabs-R-induced mRNA expression of lysosomal genes such as *Lamp1* and *Lamp2* (Figure 4C). These data suggest that Rufomycin 4–7 facilitates the lysosomal gene expression through induction of TFEB activation in macrophages during Mabs-R infection.

**FIGURE 5:**
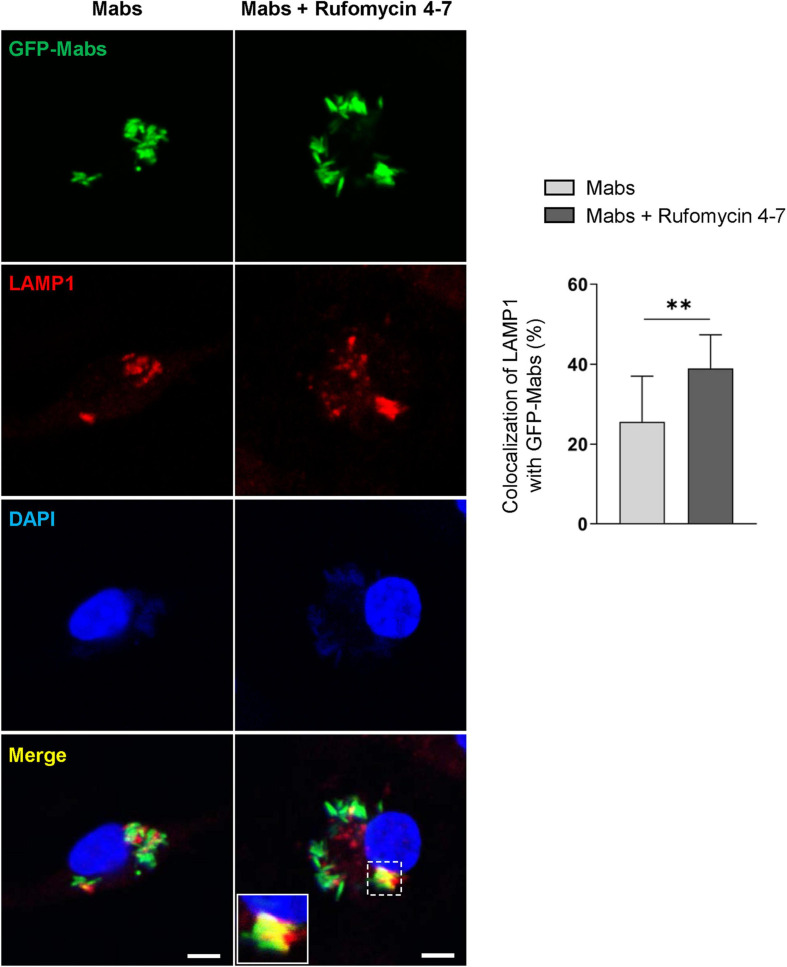
Rufomycin 4–7 increases the colocalization of phagosomes and lysosomes against GFP-Mabs infection. BMDMs were infected with GFP-Mabs (MOI = 3) for 2 h and incubated with Rufomycin 4–7 (10 μM) for 18 h in the fresh media. Cells were then stained with LAMP1 (red) and DAPI (blue) and subjected to confocal microscopy. Representative confocal images from each group (scale bar: 5 μm) and the quantitative data of colocalization of LAMP1 and GFP-Mabs were presented (*n* = 10 for each group; at least 80 cells per image). ^∗∗^*p* < 0.01. Statistical analysis was determined with unpaired *t*-test and presented as means ± SD from at least three independent experiments performed in triplicate.

Previous studies showed that the induction of intraphagosomal acidification, i.e., phagosomal colocalization with the lysosomal marker LAMP1, was essential for host defense against Mabs infection in BMDMs (Kim Y. S. et al., 2020). It was thus questioned whether Rufomycin 4–7 upregulated the colocalization of phagosomes and lysosomes. To examine this, the colocalization levels of bacterial phagosomes with lysosomes were analyzed *via* confocal imaging. Intriguingly, Rufomycin 4–7 significantly increased the colocalization of Mabs-enhanced green fluorescent protein (GFP-Mabs) phagosomes with lysosomes in BMDMs at 18 hpi (Figure 5). Collectively, these data strongly suggest that Rufomycin 4–7 significantly upregulates the lysosomal gene expression and activities during Mabs infection.

### Rufomycin 4–7 Inhibits Mitochondrial Damage and Oxidative Stresses During Mabs-R Infection

**FIGURE 6:**
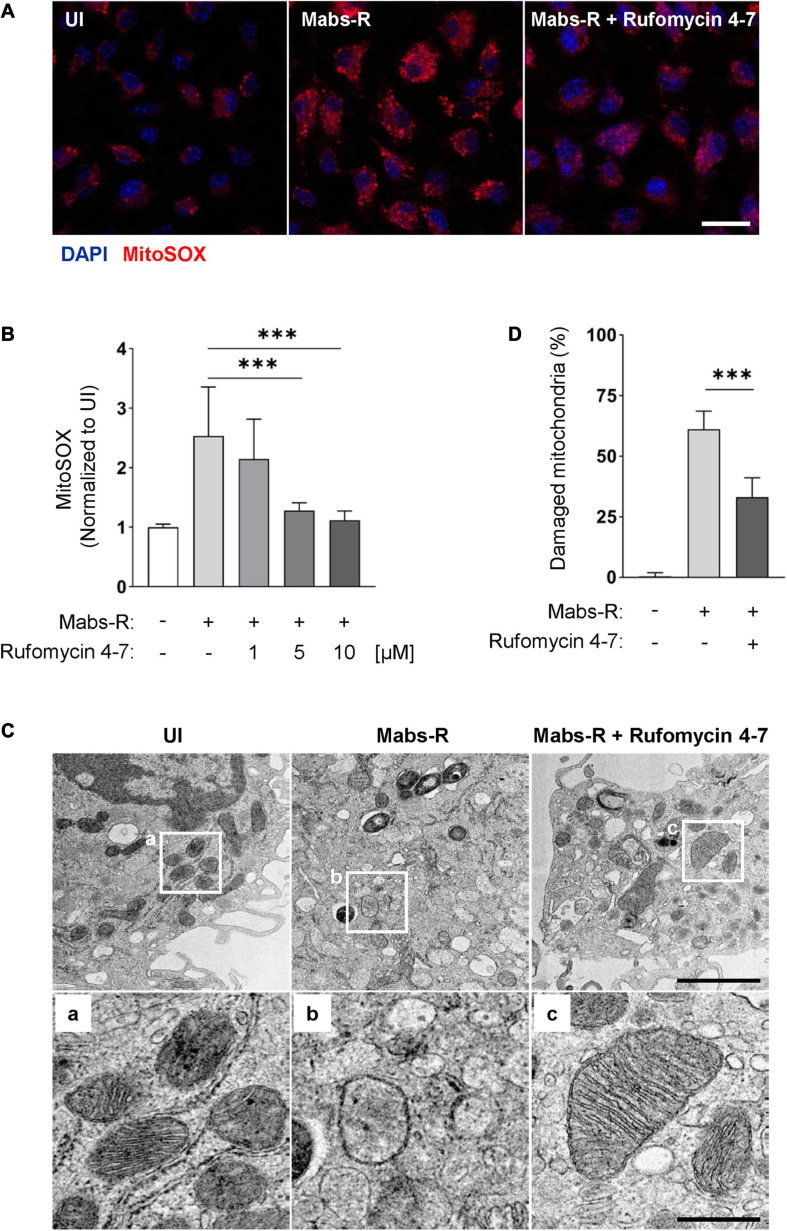
Rufomycin 4–7 ameliorates mitochondrial damage and oxidative stress during Mabs-R infection. **(A)** BMDMs were infected with Mabs-R (MOI = 3) for 2 h and further cultured with Rufomycin 4–7 (1, 5, or 10 μM) for 2 h in the fresh media. The cells were stained with MitoSOX and visualized with confocal images (scale bar: 12.5 μm). **(B)** The relative intensity level of mtROS of infected BMDMs was calculated relative to UI (*n* = 9 for each group; at least 80 cells per image). **(C)** BMDMs were infected with Mabs-R (MOI = 3) for 2 h and cultured with Rufomycin 4–7 (10 μM) for 18 h. Cells were harvested and then subjected to TEM analysis. Representative images and their magnified images (a–c) from each group are shown (scale bar: 500 nm or 1 μm). **(D)** The ratio of damaged/total mitochondria was manually calculated from at least nine EM images. ^∗∗∗^*p* < 0.001. Statistical analysis was determined with one-way ANOVA **(B,D)** and presented as means ± SD from at least three independent experiments performed in triplicate. UI, uninfected.

The increased mitochondrial oxidative stresses are associated with hyperinflammatory responses and detrimental function in antimicrobial effects during Mabs-R infection (Kim Y. J. et al., 2020). Thus, mtROS was measured in BMDMs by MitoSOX Red, a fluorescent probe for mtROS (Bulua et al., 2011). Similar to the previous findings (Kim Y. J. et al., 2020), Mabs-R infection of BMDMs led to an increase of mtROS production at 2 hpi, which was decreased with Rufomycin 4–7 treatment (Figure 6A). The relative fluorescent intensity level of MitoSOX was quantified to show that an increase of Rufomycin 4–7 dose gradually and significantly diminished the mtROS level of Mabs-R-infected BMDMs (Figure 6B).

In addition, transmission electron microscopy (TEM) analysis showed that Rufomycin 4–7 markedly ameliorated the morphological findings of mitochondrial damage, i.e., mitochondrial dilatation and mitochondrial fragmentation, in BMDMs induced by Mabs-R infection (Figure 6C). Rufomycin 4–7 treatment significantly downregulated the numbers of damaged mitochondria induced by Mabs-R infection in TEM images (Figure 6D). Moreover, tetramethylrhodamine, ethyl ester (TMRE) staining showed that disrupted mitochondrial membrane potential upon Mabs-R infection was partly recovered with Rufomycin 4–7 treatment (Supplementary Figure 4). Taken together, these data imply that Rufomycin 4–7 plays a role in the maintenance of mitochondrial homeostasis in macrophages during Mabs-R infection.

## Discussion

The present study provides evidence that Rufomycin 4–7 exhibits a dual mode of action by inducing antimicrobial responses and host defense. While Rufomycin 4–7 is a well-founded potential lead compound essential for Mtb and Mabs-S through targeting ClpC1 of mycobacteria (Choules et al., 2019), it is largely unknown whether Rufomycin 4–7 regulates host protective immune responses. The work presented here reveals that Rufomycin 4–7 plays a critical role in the suppression of proinflammatory cytokine responses, activation of lysosomal functions, and downregulation of mitochondrial damage, thus contributing to prevention of pathological responses during Mabs-R infection.

We found that Rufomycin 4–7 effectively decreased the generation of pro-inflammatory cytokines *Il1b* and *Il6* in macrophages (Figure 2 and Supplementary Figure 3) as well as the intracellular survival of type strain and two clinical isolates of Mabs-R during infection (Figure 1 and Supplementary Figure 2). Recent studies showed that both Mabs-R and Mabs-S strains resulted in exaggerated inflammatory responses in mouse lung infection models (Kim Y. J. et al., 2020; Kim Y. S. et al., 2020), although most studies reported that the Mabs-R morphotype often causes a “hyper-proinflammatory” response *in vitro* and *in vivo* and is associated with clinical severity (Rhoades et al., 2009; Roux et al., 2011). The increased virulence induced by Mabs-R depends on the production of serpentine cords, which trigger abscess formation and confer resistance to host cell phagocytosis (Bernut et al., 2014). In addition, Mabs-R, but not Mabs-S, strains enhanced inflammatory cytokines, in particular type I IFN production, through cGAS-STING-dependent pathways, to promote virulence during infection (Kim B. R. et al., 2019, 2020). Lipoproteins from Mabs-R are the major TLR2 agonists, thus inducing exaggerated inflammatory responses through TLR2 (Roux et al., 2011). Mabs-mediated TLR2 innate response is at least partly through phosphatidyl-myo-inositol mannosides in the cell wall of Mabs-R, suggesting that the underlying cell wall lipids below GPL are involved in the activation of inflammatory and innate immune responses, promoting airway inflammation (Rhoades et al., 2009). However, the maintenance of TNF/IL-8 inflammatory signaling is critical for protective immunity against Mabs infection, because loss of TNFR1 function in zebrafishes increased mortality and unrestricted intramacrophage bacterial growth, regardless of Mabs-R or Mabs-S strains (Bernut et al., 2016). Combined with these results and ours, the manipulation of the balance to prevent either excessive or deprived inflammatory response may contribute to host immune protection during Mabs infection.

Moreover, Mabs and other mycobacteria functions act as either inducers of or evaders from intracellular signaling pathways, including NF-κB and MAPK pathways, in innate immune responses during infection (Roach and Schorey, 2002; Schorey and Cooper, 2003; Jo et al., 2007). Our data suggest that the Mabs-R variant is not a strong inducer of a variety of intracellular signaling pathways, but robustly triggers the signaling pathway of p38 MAPK, a key serine and threonine protein kinase that is critical for inflammatory responses in macrophages (Yang et al., 2014). Future studies will be geared toward understanding how Rufomycin 4–7 regulates p38 MAPK pathway activation in macrophages during Mabs-R infection. Recently, it was shown that peroxisome proliferator-activated receptor α (PPARα) activation is required for the activation of TFEB, which is essential for antimicrobial responses and intraphagosomal acidification, i.e., phagosomal colocalization with the lysosomal marker LAMP1, during Mabs-S infection (Kim Y. S. et al., 2020). In addition, nuclear translocation and activation of TFEB, which are regulated by sirtuin 3 and PPARα pathways, are crucial for antimicrobial host defense against Mtb infection (Kim et al., 2017; Kim T. S. et al., 2019). However, it is largely unknown how TFEB expression and nuclear translocation are modulated during Mabs-R infection. In the present work, Mabs-R infection of macrophages markedly decreased the expression and nuclear translocation of TFEB. These results partly agree with recent data showing that pathogens such as *Salmonella* and *Staphylococcus aureus* inhibited the TFEB expression and activation, thereby blocking the lysosomal functions and escape from host defense during infection (Rao et al., 2020). Interestingly, Rufomycin 4–7 significantly recovered the Mabs-R-mediated decrease of TFEB expression in macrophages. In addition, the rescue of TFEB activation by Rufomycin 4–7 led to an activation of TFEB-downstream autophagy/lysosomal genes and enhanced colocalization between GFP-Mabs phagosomes and lysosomes. Meanwhile, Roux et al. (2016) reported the distinct effect of Mabs-R and Mabs-S in blocking autophagy. They showed the Mabs-S was more capable in restricting intraphagosomal acidification and induced less autophagy than Mabs-R (Roux et al., 2016). Thus, it would be the future target to compare the different aspects between Mabs-R and Mabs-S in terms of Rufomycin 4–7-induced autophagy and understand how Rufomycin 4–7 activates TFEB nuclear translocation and upregulates the TFEB-related gene subsets under Mabs infection. It was further found that Rufomycin 4–7 inhibits mtROS in macrophages during Mabs-R infection. In addition, it was recently found that innate immune defense induced by sirtuin 3 activation is at least partly mediated through the suppression of excessive mitochondrial damage during Mabs-R infection (Kim Y. J. et al., 2020). Together with the current findings, it is also plausible that blocking mitochondrial damage during antibiotic treatment may contribute to integral host responses during chemotherapy against chronic infection.

Treatment of NTM infections is challenging, because it requires multidrug regimens for at least 18 months and is often associated with serious side effects and drug resistance (Falkinham, 2018; Sethiya et al., 2020). This explains the dire need for the development of new drugs that can promote both host immune responses and unfold antimicrobial activity against Mabs-R infection. With respect to these findings, the present outcomes strongly support the potential of developing Rufomycin 4–7 into a drug candidate that possesses dual effects upon host and pathogens against Mabs-R infection, in addition to its anti-Mtb treatment potential.

## Materials and Methods

### Preparation of Crude Extract

*Streptomyces* sp. MJM3502 was cultured in modified GSS medium at 28°C. Crude extracts were obtained as previously reported (Zhou et al., 2020). Briefly, cultured whole broth was mixed with methanol, filtered, and concentrated. The concentrated solution was dissolved in methanol/water solution and partitioned with ethyl acetate (EtOAc). The EtOAc layer was evaporated to dryness to yield a yellowish solid extract. Then the extract was subjected to silica column chromatography, and the rufomycin-enriched fractions were finally collected by elution with hexane and EtOAc.

### Rufomycin 4–7 Purification

A rufomycin-enriched fraction (14 g) of an extract of the *Streptomyces* sp. MJM3502 was divided into three portions (A–C), which were further chromatographed on different adsorbents. Portion A (4.7 g) was separated on silica gel using a gradient elution of chloroform/methanol (200:1, 100:1, 50:1, 20:1, and 10:1) to give 12 fractions (A1–A12). Portions B (4.2 g) and C (4.1 g) were separated with Bakerbond C3 and Diaion HP-21, respectively. Using gradient elution with methanol/water (40–95%), seven fractions (B1–B7) and eight fractions (C1–C8) were obtained, respectively. HPLC analysis revealed that fractions A5–A7, B4–B6, and C6–C7 were enriched in Rufomycin 4–7. They were combined and separated by column chromatography on silica gel using gradient elution with hexane/acetone (5:1, 4:1, 3:1, 2:1, 1:1, and 1:2). Collectively, this yielded 4.4 g of Rufomycin 4–7. In HPLC-DAD purity analysis under 190 nm, the purity of Rufomycin 4–7 used in bioassay was 99.3%. Moreover, the ratio of each rufomycin in the sample is shown as Rufomycin 4:5:6:7 = 41.5:1.4:55.0:1.4%, respectively. In NMR analysis, the integral ratio of 24-CH_2_ signals in each rufomycin 4 and 6 was 43.2–56.8% (Supplementary Figure 5).

### Mice

C57BL/6 wild-type mice were purchased from Samtako Bio Korea (Gyeonggi-do, South Korea), and 6–8-week-old females were used in the experiments. All animal experiments conducted in this study were approved by the Institutional Research and Ethics Committee at Chungnam National University School of Medicine (202009A-CNU-155; Daejeon, South Korea). All animal-related procedures were conducted in accordance with the guidelines of the Korean Food and Drug Administration.

### Mycobacterial Strains and Culture

The ATCC19977 strain of Mabs-R, clinical strain KMRC-00800-00018 (#18) and KMRC-00800-00019 (#19) of Mabs-R, KMRC-00136-13018 strain of Mmass-R, and GFP-Mabs were used for this study. Mmass-R and three different strains of Mabs-R were kindly provided by Dr. Whang from the Korean Institute of Tuberculosis. Mycobacteria were incubated at 37°C with shaking in Middlebrook 7H9 broth (BD Biosciences, Franklin Lakes, NJ, United States, 271310) containing 1% glycerol, 0.25% Tween 80, and 10% OADC until the mid-logarithmic phase (OD = 0.6) and harvested by centrifugation at 3,000 rpm for 30 min. The Mabs-R or Mmass-R clumps were separated into single cells using a sonicator in phosphate-buffered saline supplemented with 0.1% Tween 80 (PBST) and stored at −80°C. GFP-Mabs was obtained from Gyeongsang National University and cultured as previously described (Kim Y. S. et al., 2020).

### Isolation of BMDMs

Bone marrow cells were collected from femur and tibia of C57BL/6 wild-type mice (6–8 weeks old) and cultured as previously described (Kim Y. J. et al., 2020). Briefly, cells were differentiated to BMDMs in Dulbecco’s modified Eagle’s medium (DMEM; Lonza, Walkersville, MD, United States) containing 10% fetal bovine serum (FBS; Gibco, Grand Island, NY, United States) and antibiotics (Lonza, 17-745E) in the presence of 25 μg/ml macrophage colony-stimulating factor (M-CSF; R&D Systems, Minneapolis, MN, United States) for 4–5 days in a 37°C incubator.

### Colony-Forming Unit Assays

For *in vivo* colony-forming unit (CFU) assays, Mabs-R was administered to mice intranasally (i.n.; 1 ×10^6^ CFU/mouse) and the lungs from infected mice were collected at 21 days, following the experimental plan. The lung tissues were homogenized using a homogenizer (OMNI TH; Omni International, Kennesaw, GA, United States) in PBST and were serially diluted with PBST. For *in vitro* CFU assays, BMDMs were infected for 2 h with Mabs-R, washed with PBS, and further harvested in freshly changed media. At 1 dpi, the cells were lysed with 200 μl of tertiary distilled water for 30 min to release intracellular bacteria and then serially diluted with PBST. The dilutions of the homogenized lung tissues or lysed cells were plated on Middlebrook 7H10 agar petri dishes. Bacterial colonies were counted after 3 days of incubation at 37°C.

### Histology and Immunohistochemistry

Lungs were collected from mice infected with Mabs-R for 21 days. The tissues were fixed in 10% formalin and embedded in paraffin wax. The sectioned tissues (4 μm) were stained with hematoxylin and eosin (H&E). H&E-stained images were scanned using the PANNORAMIC 300 Flash DX (3DHISTECH, Budapest, Hungary). To quantify the percentage of inflamed area, the red-stained area with high threshold from three lungs retrieved from sacrificed mice was measured and divided by each lung area using FIJI software. For immunohistochemical (IHC) staining, lung paraffin blocks were sectioned and stained with antibody specific for anti-mouse Ly6G (Bio X Cell, Lebanon, NH, United States, BP0075-1). For analysis of neutrophils, anti-Ly6G-stained lung tissue images were taken with a confocal laser scanning microscope. The relative integrated intensity of Ly6G-stained cells was measured from random lung tissue images using MetaMorph Advanced Imaging acquisition software version 7.8 (Molecular Devices, San Jose, CA, United States).

### Immunofluorescence Analysis

After the appropriate treatment, media were removed and cells on coverslips were washed with PBS three times and fixed with 4% paraformaldehyde for 10 min. The cells were permeabilized with 0.25% Triton X-100 (Sigma-Aldrich, St. Louis, MO, United States) for 10 min and incubated overnight with the appropriate primary antibodies as follows at 4°C. Anti-TFEB antibody (A303-673A) was purchased from Invitrogen (Thermo Fisher Scientific, Carlsbad, CA, United States) for the TFEB staining, and anti-LAMP1 (sc-5570) was purchased from Santa Cruz Biotechnology (Dallas, TX, United States) to indicate lysosomes. The cells were washed with PBS three times and incubated with the secondary antibody for 2 h at room temperature. Nuclei were stained with DAPI for 5 min and then mounted with Fluoromount-G (SouthernBiotech, Birmingham, AL, United States, 0100-01). Fluorescent images were acquired using the TCS SP8 confocal microscope (Leica, Wetzlar, Germany). Quantification of TFEB-nuclear translocation was performed by manual calculation, and the percentage of lysosomes colocalized with GFP-Mabs was analyzed using MetaMorph Advanced Imaging acquisition software version 7.8 (Molecular Devices, San Jose, CA, United States) by calculating green signals (GFP-Mabs) overlapping red signals (LAMP1). Meanwhile, to observe the mitochondria of living cells, BMDMs were cultured with MitoSOX Red Mitochondrial Superoxide Indicator (Invitrogen, United States, M36008) for 20 min. The cells were washed three times and stained with DAPI. Fluorescent images were acquired using the TCS SP8 confocal microscope (Leica, Wetzlar, Germany), and the relative integrated intensity of the red signals was determined using FIJI software to measure the mitochondrial ROS level.

### Drug Susceptibility Test of Mabs-R Clinical Isolates

Rufomycin 4–7 and linezolid susceptibilities of Mabs-R clinical strains were tested by measuring the MIC using the broth microdilution concentration method (BMCM). The method was referred to as the CLSI guideline, except for the composition of the medium and the concentration of Rufomycin 4–7. To prepare the MIC test plate, Rufomycin 4–7 and linezolid were diluted twofold serially in 11 wells of round-bottom 96-well microplates from 32 to 0.03125 μg/ml for Rufomycin 4–7 and from 64 to 0.0625 μg/ml for linezolid with 0.1 ml of 7H9 medium containing 10% of OADC supplement (BD, Franklin Lakes, NJ, United States). Mabs clinical isolates from the Korea Mycobacterium Resource Center of the Korean Institute of Tuberculosis were cultured in 7H10 agar medium containing 10% of OADC, then inoculated in 10 ml of 7H9-OADC liquid medium and incubated at 37°C for 1 week. After the bacterial growth was confirmed, the Mabs strains were diluted as much as 0.5 McFarland standard in 20 ml of 7H9-OADC containing 50 μg/ml of 2,3-diphenyl-5-thienyl-(2)-tetrazolium chloride (STC; TCI, Tokyo, Japan) for the mycobacterial staining and inoculated 100 μl for each well of prepared MIC microplates. The MICs of Mabs clinical strains were determined on the fourth day and the seventh day after the incubation.

### RNA Extraction and Quantitative Real-Time PCR Analysis

Total RNA was isolated from cells using TRIzol (Thermo Fisher Scientific, Waltham, MA, United States, 15596026), following the manufacturer’s protocol. Complementary DNA (cDNA) from total RNA was synthesized by reverse transcription using Reverse Transcriptase Premix (Elpis Biotech, EBT-1515, Daejeon, South Korea). Quantitative real-time PCR (qRT-PCR) was performed on the Rotor-Gene Q 2plex system (Qiagen, 9001620), using primers for the indicated genes and SYBR Green Master Mix (Qiagen, 218073) following the manufacturer’s instructions. Relative expression levels of mRNA were analyzed using the 2^–ΔΔCt^ method, with normalization to actin gene expression. The primer sequences used are shown in Supplementary Table 2.

### ELISA Analysis

The concentrations of proinflammatory cytokines TNF-α (558534), IL-6 (555240), and IL-1β (559603) in cell culture supernatants were measured using a Mouse BD OptEIA Set ELISA Kit (BD Biosciences, San Diego, CA, United States) according to the manufacturer’s protocol.

### Immunoblot Analysis

BMDMs were lysed in RIPA buffer (150 mM sodium chloride, 1% Triton X-100, 0.1% SDS, 1% sodium deoxycholate, 50 mM Tris–HCl at pH 7.5, and 2 mM EDTA) containing protease and phosphatase inhibitor cocktail (Roche, Mannheim, Germany). The cell extracts were mixed with Protein 5× Sample Buffer (ELPIS BIOTECH, EBA-1052) and boiled for 10 min. The protein extracts were subjected to SDS-PAGE and transferred to PVDF membranes (Millipore, Burlington, MA, United States). The membranes were blocked with 5% skim milk in Tris-buffered saline supplemented with 0.1% Tween 20 (TBST) for 1 h at RT and then incubated O/N at 4°C with the following primary antibodies. Anti-β-actin (sc-47778) was purchased from Santa Cruz Biotechnology (Dallas, TX, United States). Anti-phospho-NF-κB (3033), anti-phospho-ERK1/2 (9101), anti-phospho-SAPK/JNK (4668), anti-phospho-p38-MAPK (9211), or anti-phospho-Akt (4060) were purchased from Cell Signaling Technology (Danvers, MA, United States). After washing with TBST and incubating for 1 h with anti-mouse IgG (7076) or anti-rabbit IgG (7074) secondary antibodies (Cell Signaling Technologies) at RT, protein signals were visualized using enhanced chemiluminescence solution (Millipore, WBKL S0500) and detected using a UVitec Alliance mini-chemiluminescence device (UVitec, Rugby, United Kingdom). The densitometric values were calculated using ImageJ software, and data were normalized to β-actin loading control.

### TEM Analysis

BMDMs were cultured in a 60-mm cell culture dish at least in duplicate and properly treated according to the experimental design. After removing the culture medium, the cells were fixed with 3% glutaraldehyde in PBS. The cells were scraped using a scraper, carefully gathered, and fixed with 1% osmium tetroxide on ice for 2 h and washed with PBS. The cells were dehydrated in propylene oxide and ethanol series at 4°C, then embedded in Embed-812 solution and polymerized in 70°C oven for 24 h. The sections from polymerized blocks were mounted on 150 mesh copper grids, stained with 4% uranyl acetate and lead citrate for 7 min. The samples were scanned using Bio-HVEM system (JEM-1400 Plus and HEM-1000 BEF; JEOL Ltd., Tokyo, Japan).

### TMRE Analysis

Mabs-R-infected peritoneal macrophages were incubated with 200 nM TMRE (Invitrogen, T669) at 37°C in the dark. After 20 min, the cells were measured by flow cytometry using the FL2 channel (582 nm). Data were analyzed using the FlowJo software.

### Statistical Analysis

Statistical analysis was performed using Prism (GraphPad Software, v8.4.0.671). The results were appropriately processed by unpaired *t*-test or ANOVA and are described in each figure legend. The results of *in vivo* experiments are presented as means ± standard error of the mean (SEM) and the data of *in vitro* assays are expressed as means ± standard deviation (SD); ^∗^*p* < 0.05, ^∗∗^*p* < 0.01, and ^∗∗∗^*p* < 0.001 were considered indicative of statistical significance.

## Data Availability Statement

The raw data supporting the conclusions of this article will be made available by the authors, without undue reservation.

## Ethics Statement

The animal study was reviewed and approved by the Institutional Research and Ethics Committee, Chungnam National University School of Medicine.

## Author Contributions

CRP executed most of the data processing and analysis. SP, YJK, JKK, SMJ, S-HL, and JW participated in the experiments and data analysis. JCh, JC, GS, and S-NC performed the purification, characterization, and quality control of the compound used in this study. CRP, SP, and E-KJ wrote the bulk of the manuscript, and critically reviewed by J-WS, GS, JM, GFP, and SF. E-KJ and SC guided and supervised the work. All authors reviewed and edited the manuscript.

## Conflict of Interest

The authors declare that the research was conducted in the absence of any commercial or financial relationships that could be construed as a potential conflict of interest.

## Publisher’s Note

All claims expressed in this article are solely those of the authors and do not necessarily represent those of their affiliated organizations, or those of the publisher, the editors and the reviewers. Any product that may be evaluated in this article, or claim that may be made by its manufacturer, is not guaranteed or endorsed by the publisher.
