# Supplementary material for: Rufomycin Exhibits Dual Effects Against Mycobacterium abscessus Infection by Inducing Host Defense and Antimicrobial Activities
Source: Front Microbiol. 2021 Aug 10;12:695024. doi: 10.3389/fmicb.2021.695024 (PMC8383285; doi:10.3389/fmicb.2021.695024)
Supplement: Supplementary file 1 [file Data_Sheet_1.doc]

**
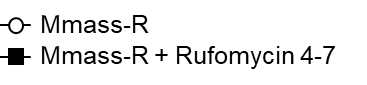
**

**
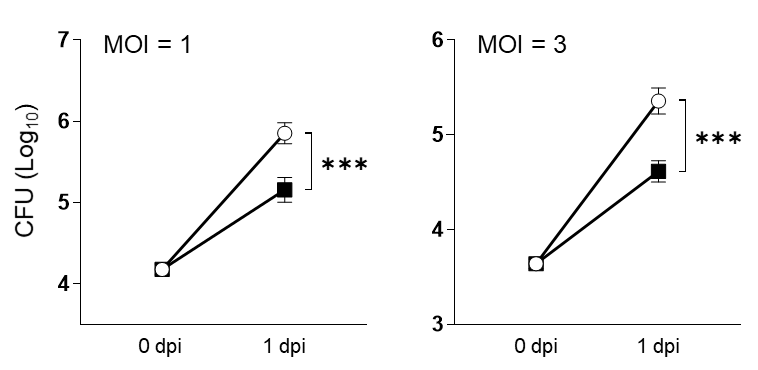
**

**Supplementary Figure 1. Rufomycin 4-7 treatment increases antimicrobial effects against Mmass-R infection *in vitro*.** BMDMs were infected with Mmass-R (MOI = 1 or 3) for 2 h and then incubated with sc or Rufomycin 4-7 (10 μM) in the fresh media. Intracellular survival of Mmass-R was determined by CFU assay at 0 and 1 dpi. ****p* < 0.001. Two-way ANOVA. Data are combined results (mean ± SD) from three independent experiments. dpi, days-post infection.

**
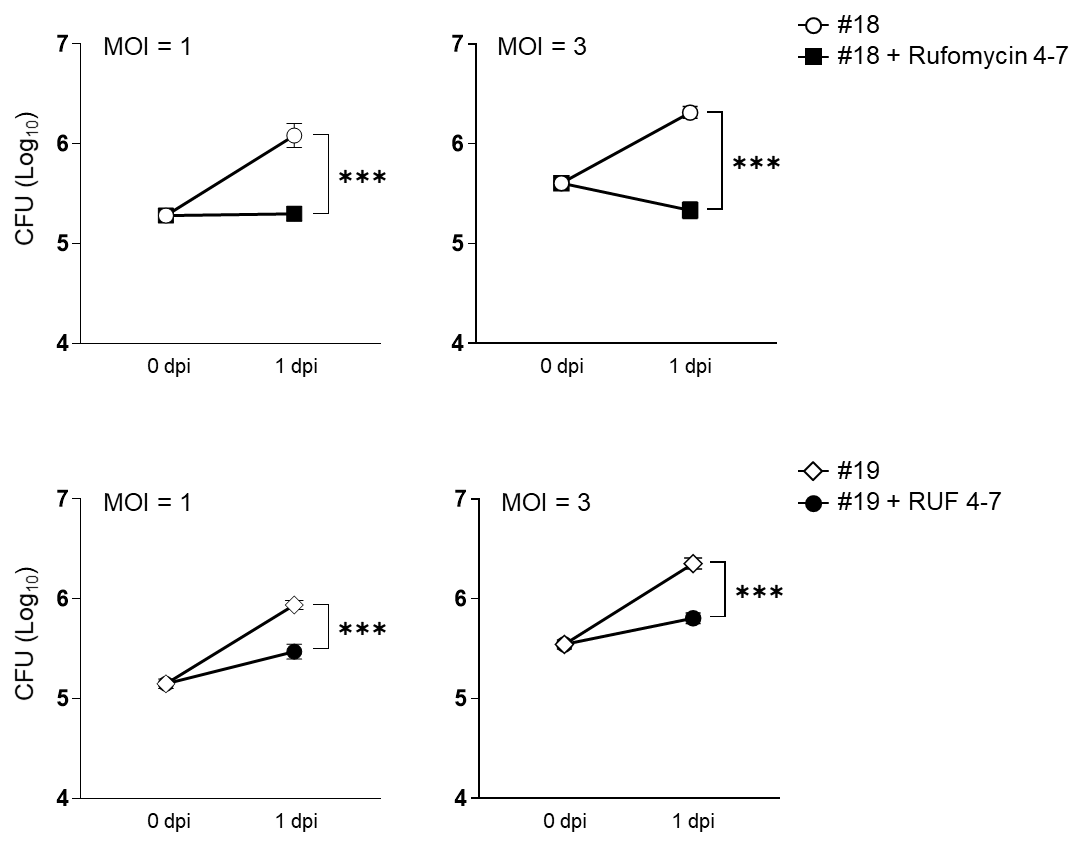
**

**Supplementary Figure 2. Rufomycin 4-7 treatment increases antimicrobial effects against rough type of clinical isolates infection *in vitro*.** BMDMs were infected with #18 or #19 (MOI = 1 or 3) for 2 h and then incubated with sc or Rufomycin 4-7 (10 μM) in the fresh media. Intracellular survival of mycobacteria was determined by CFU assay at 0 and 1 dpi. ****p* < 0.001. Two-way ANOVA. Data are combined results (mean ± SD) from three independent experiments. dpi, days-post infection


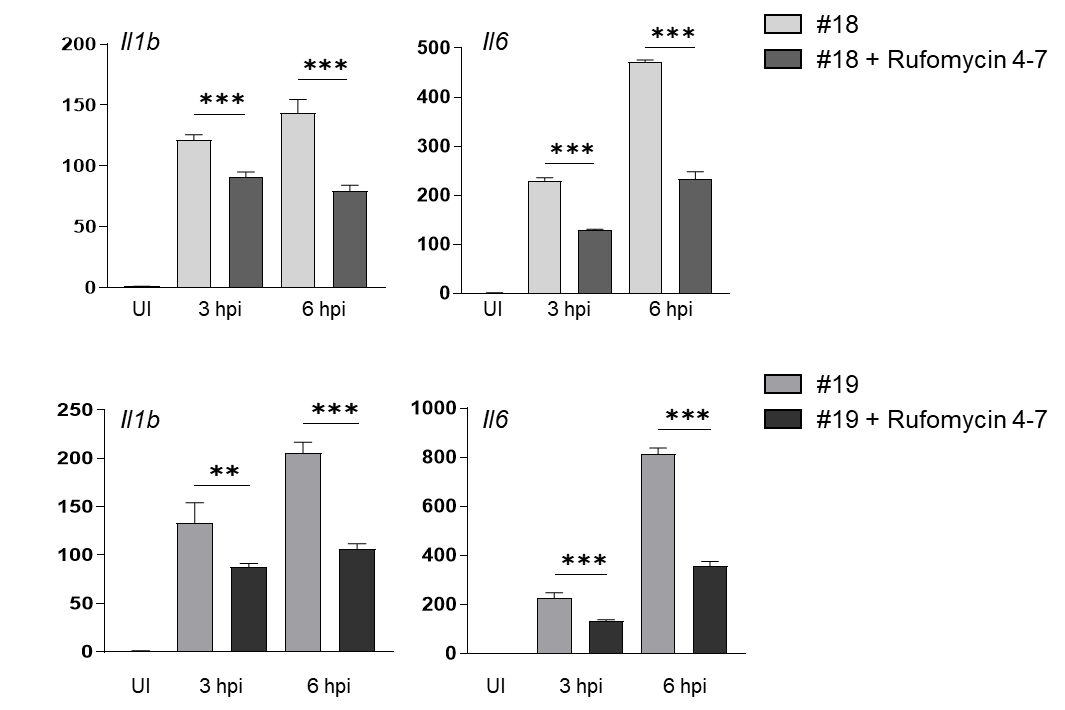


**Supplementary Figure 3. Rufomycin 4-7 regulates the expression of inflammatory cytokines during rough type of clinical** **isolates** **infection.** BMDMs were infected with #18 or #19 (MOI = 3) for 2 h, then cultured with sc or Rufomycin 4-7 (10 μM) in the freshly changed media. The cells were harvested at the indicated times and subjected to quantitative real-time PCR (qRT-PCR) analysis to measure the expression of proinflammatory cytokine genes. ***p* < 0.01, ****p* < 0.001. Statistical analysis was determined with one-way ANOVA and presented as means ± SD from at least three independent experiments performed in triplicate. ns, not significant; UI, uninfected; hpi, hours-post infection.


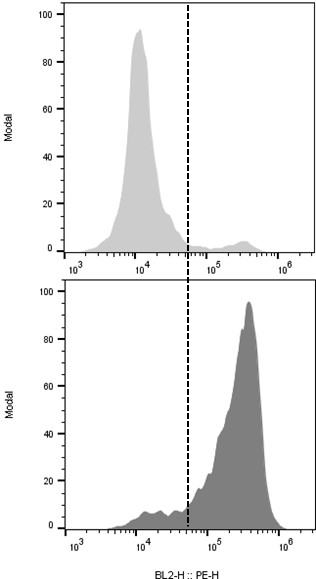


**
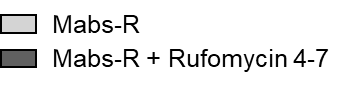
**

**
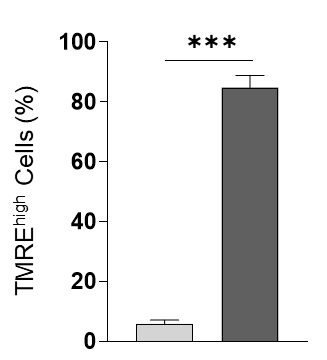
**

**Supplementary Figure 4. Effects of Rufomycin 4-7 on Mabs-R-induced mitochondrial dysfunction in peritoneal macrophages.** Peritoneal macrophages were infected with Mabs-R (MOI=3) for 5 h, followed by treatment with sc or Rufomycin 4-7 (10μM) for 3 h. Mitochondrial membrane potentials (as measured by TMRE) were analyzed by flow cytometry. ****p* < 0.001. Unpaired T-test.

**
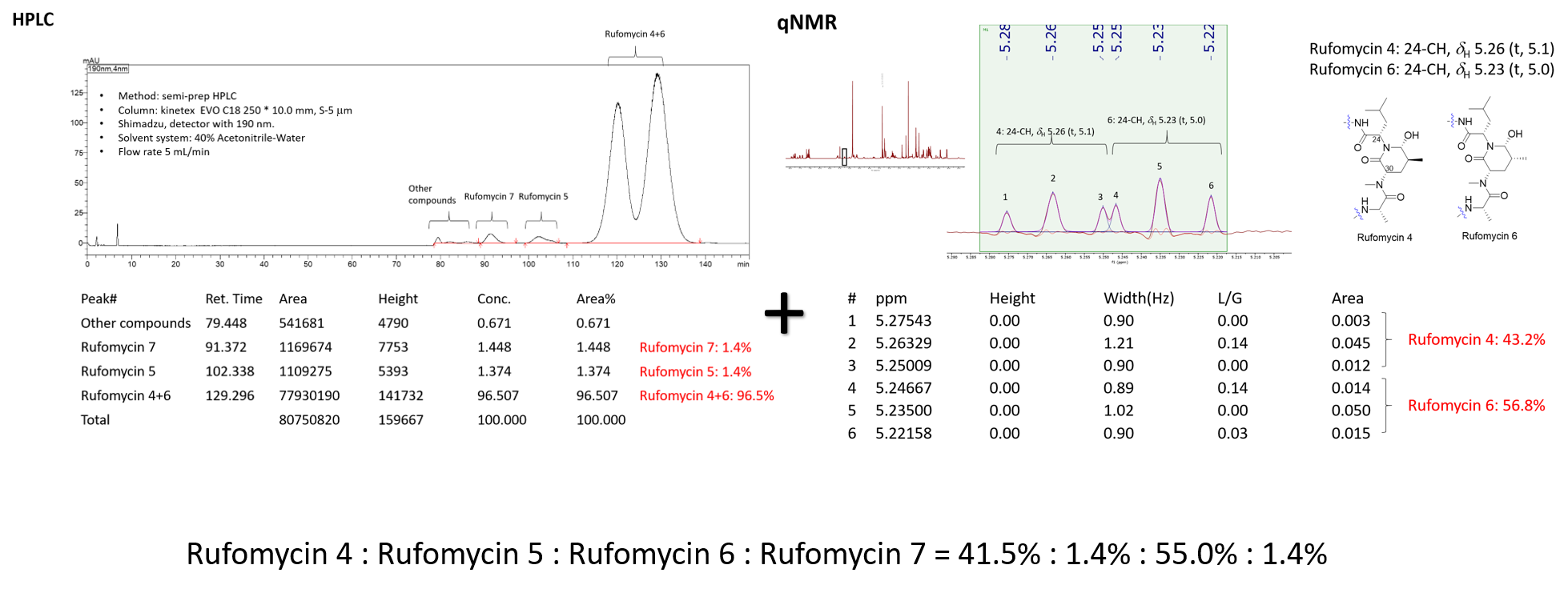
**

**Supplementary Figure 5. HPLC and qNMR data for determining the ratio of four rufomycins.** In HPLC analysis, the column (Kinetex EVO C18, 250 × 10.0 mm, S-5 μm) was used with 40% Acetonitrile-Water as solvent system and 5 ml/min as flow rate. The data was analyzed using LabSolutions software. The ^1^H NMR spectrum of Rufomycin 4-7 was acquired on a JEOL (JEOL Resonance Inc., Peabody, MA, USA) ECZ 400S spectrometer, and MestReNova software was used for data analysis.

**Supplementary Table 1. Minimal inhibitory concentration (MIC) values of Rufomycin 4-7 and linezolid among clinical isolates and type strain of Mabs-R.** The morphologies of Mabs strains were identified into rough type according to the texture of their colonies formed after culturing in 7H10 solid medium for 1 week. MICs of Rufomycin 4-7 in 3 rough-type strains including rough variant of ATCC19977 type strain were measured, and linezolid MICs were also measured for the reference.

| **Strain No.** | **Morphology** | **Rufomycin 4-7**  **μg/ml** | **Linezolid**  **μg/ml** |
| --- | --- | --- | --- |
| ATCC 19977 | Rough | 4 | 8 |
| #18 | Rough | 0.5 | 4 |
| #19 | Rough | 4 | 4 |

**Supplementary Table 2.** **Mouse primer sequences used for qRT-PCR analysis**

| Genes | Primer | Sequences |
| --- | --- | --- |
| Mouse |  |  |
| *Actin* | Forward  Reverse | 5′-AGG GTG TAA AAC GCA GCT CA-3′  5′-CCA CCA TGT ACC CAG GCA TT-3′ |
| *Tnf* | Forward  Reverse | 5′-CCCACGTCGTAGCAAACCAC-3′  5′-GCAGCCTTGTCCCTTGAAGA-3′ |
| *Il1b* | Forward  Reverse | 5′-TGA CGG ACC CCA AAA GAT GA-3′  5′-AAA GAC ACA GGT AGC TGC CA-3′ |
| *Il6* | Forward  Reverse | 5′-TAC CAC TTC ACA AGT CGG AGG C-3′  5′-CTG CAA GTG CAT CAT CGT TGT TC-3′ |
| *Cxcl2* | Forward  Reverse | 5′-GAA GTC ATA GCC ACT CTC AAG G-3′  5′-CCT CCT TTC CAG GTC AGT TAG C-3′ |
| *Cxcl5* | Forward  Reverse | 5′-GCA CTC GCA GTG GAA AGA AC-3′  5′-CGT GGG TGG AGA GAA TCA GC-3′ |
| *Ccl2* | Forward  Reverse | 5′-TCT CTC TTC CTC CAC CAC CAT G-3′  5′-GCG TTA ACT GCA TCT GGC TGA-3′ |
| *Ccl4* | Forward  Reverse | 5′-TCT GTG CAA ACC TAA CCC CG-3′  5′-GAG GGT CAG AGC CCA TTG GT-3′ |
| *Il12p40* | Forward  Reverse | 5′-TTG AAC TGG CGT TGG AAG CAC G-3′  5′-CCA CCT GTG AGT TCT TCA AAG GC-3′ |
| *Il10* | Forward  Reverse | 5′-GCT CTT GCA CTA CCA AAG CC-3′  5′-CTG CTG ATC CTC ATG CCA GT-3′ |
| *Tfeb* | Forward  Reverse | 5′-CGC CTG GAG ATG ACT AAC AAG C-3′  5′-GGC AAC TCT TGC TTC ACC ACC T-3′ |
| *Uvrag* | Forward  Reverse | 5′-GAC TTT GGA ATA ATG CCG GAT CG-3′  5′-CAG CCC ATC CAG GTA GAC TTT-3′ |
| *Beclin1* | Forward  Reverse | 5′-CAG CCT CTG AAA CTG GAC ACG A-3′  5′-CTC TCC TGA GTT AGC CTC TTC C-3′ |
| *Gabarap* | Forward  Reverse | 5′-GGT CCC GGT GAT AGT GGA AAA A-3′  5′-AAC AAG GCA TCT TCA GCA CG-3′ |
| *Rab7* | Forward  Reverse | 5′-GAG CGG ACT TTC TGA CCA AGG A-3′  5′-CAA TCT GCA CCT CTG TAG AAG GC-3′ |
| *Lamp1* | Forward  Reverse | 5′-CAG CAC TCT TTG AGG TGA AAA AC-3′  5′-CCA TTC GCA GTC TCG TAG GTG-3′ |
| *Lamp2* | Forward  Reverse | 5′-GAG CAG GTG CTT TCT GTG TCT AG-3′  5′-GCC TGA AAG ACC AGC ACC AAC T-3′ |
